# A phenomenological model of proton FLASH oxygen depletion effects depending on tissue vasculature and oxygen supply

**DOI:** 10.3389/fonc.2022.1004121

**Published:** 2022-11-28

**Authors:** Wei Zou, Haram Kim, Eric S. Diffenderfer, David J. Carlson, Cameron J. Koch, Ying Xiao, BoonKeng K. Teo, Michele M. Kim, James M. Metz, Yi Fan, Amit Maity, Costas Koumenis, Theresa M. Busch, Rodney Wiersma, Keith A. Cengel, Lei Dong

**Affiliations:** Department of Radiation Oncology, Perelman School of Medicine, University of Pennsylvania, Philadelphia, PA, United States

**Keywords:** FLASH effect, oxygen depletion, proton, vasculature, hypoxic reduction factor

## Abstract

**Introduction:**

Radiation-induced oxygen depletion in tissue is assumed as a contributor to the FLASH sparing effects. In this study, we simulated the heterogeneous oxygen depletion in the tissue surrounding the vessels and calculated the proton FLASH effective-dose-modifying factor (FEDMF), which could be used for biology-based treatment planning.

**Methods:**

The dose and dose-weighted linear energy transfer (LET) of a small animal proton irradiator was simulated with Monte Carlo simulation. We deployed a parabolic partial differential equation to account for the generalized radiation oxygen depletion, tissue oxygen diffusion, and metabolic processes to investigate oxygen distribution in 1D, 2D, and 3D solution space. Dose and dose rates, particle LET, vasculature spacing, and blood oxygen supplies were considered. Using a similar framework for the hypoxic reduction factor (HRF) developed previously, the FEDMF was derived as the ratio of the cumulative normoxic-equivalent dose (CNED) between CONV and UHDR deliveries.

**Results:**

Dynamic equilibrium between oxygen diffusion and tissue metabolism can result in tissue hypoxia. The hypoxic region displayed enhanced radio-resistance and resulted in lower CNED under UHDR deliveries. In 1D solution, comparing 15 Gy proton dose delivered at CONV 0.5 and UHDR 125 Gy/s, 61.5% of the tissue exhibited ≥20% FEDMF at 175 μm vasculature spacing and 18.9 μM boundary condition. This percentage reduced to 34.5% and 0% for 8 and 2 Gy deliveries, respectively. Similar trends were observed in the 3D solution space. The FLASH versus CONV differential effect remained at larger vasculature spacings. A higher FLASH dose rate showed an increased region with ≥20% FEDMF. A higher LET near the proton Bragg peak region did not appear to alter the FLASH effect.

**Conclusion:**

We developed 1D, 2D, and 3D oxygen depletion simulation process to obtain the dynamic HRF and derive the proton FEDMF related to the dose delivery parameters and the local tissue vasculature information. The phenomenological model can be used to simulate or predict FLASH effects based on tissue vasculature and oxygen concentration data obtained from other experiments.

## Introduction

With more than 50% of cancer patients receiving radiation therapy, accurate dose delivery and treatment planning and delivery techniques to maximize normal tissue sparing are critical tasks for radiation oncology (2). Recently, there is increasing evidence that ultra-high dose rate (UHDR) radiation can provide notable FLASH normal tissue sparing without affecting tumor control probability (3–8). Considering that clinical radiotherapy outcomes are typically limited by normal tissue radiotoxicity, the FLASH sparing effect, if realized, can have a profound impact on future radiotherapy development. Multiple review articles on the topic have pointed out the potential of applying ultra-high dose rate radiotherapy to achieve better radio-therapeutic outcomes (9–13). As part of the effort to incorporate FLASH into current radiotherapy practice, UHDR electron treatments of human (14) and animal patients (6, 15) with superficial lesions were performed. Compared with electron radiotherapy, proton radiotherapy provides greater dose conformality and the ability to treat deep-seated tumors (16), thus an attractive modality to adopt FLASH deliveries. Recent technical developments have demonstrated that a commercial proton therapy system can be modified to achieve ultra-high dose rates (8, 17). Certain FLASH dose rate effects with the proton beams were also observed in cells and animal models (4, 8, 18, 19).

To date, the biological mechanisms of FLASH normal tissue radioprotection effect remain unclear. In published small animal UHDR experiments, multiple levels of dose and dose rates were delivered. Although most studies observed the FLASH sparing effects, a few studies have not reproduced similar findings (20, 21), or the effects were limited (4, 18). There have been discussions on a potential threshold for FLASH dose, dose rate, or a combination of both (12). A FLASH dose-modifying factor was summarized for various experiments with different experiment endpoints (11). Multiple FLASH mechanisms have been proposed. Among those, the mechanism of radiation-introduced oxygen depletion with ultra-high dose rate has gained the most popularity (12, 13, 22–25). Support for the oxygen depletion mechanism comes from multiple earlier cell (26–28) and animal (10, 29, 30) radiation studies under aerobic/normoxic and hypoxic environment where decreased oxygen tension decreases radiosensitivity. In terms of the cell level radiotoxicity, oxygen tension decreases as a result of radiochemical reactions, consequently impeding oxygen-mediated fixation of DNA damage (31, 32). In addition, oxygen depletion reduces the yields of reactive oxygen species (ROS), resulting in a radio-protective effect (5, 22). Spitz et al. (22) discussed the organic redox reactions, including the labile metal ion oxidative chain reactions that cause a substantial amount of oxygen depletion compared to water radiolysis alone. Recently, Labarbe et al. (33) quantitatively modeled the kinetics of these main reactions and differential ROS production under different dose rates. Multiple oxygen depletion/replenish models were proposed in order to quantify the oxygen effects under FLASH (24, 34, 35). These models have similar components that include radiation-introduced oxygen depletion, oxygen replenishment from the nearby vasculature, and oxygen diffusion from nearby tissues. Moreover, these studies used the oxygen enhancement ratio (OER) based on the Alper–Howard–Flanders (AHF) model (36) to examine the biological effects under UHDR deliveries.

Oxygen enhancement ratio (OER) has been proposed to quantify the radio-protection effect under reduced oxygen tension, such as in tumor hypoxia environment. It is defined as the dose ratio at anoxia and physiological oxygen levels to achieve the same biological endpoint (37) and can be used to compare the tissue radiosensitivity at various oxygen levels. Compared to photon beam, the characteristic Bragg peak of a particle beam at the end of the beam range is associated with an increase in linear energy transfer (LET), resulting in increased relative biological effectiveness (RBE) (38). The increase in RBE results from an increased initial yield of DNA double-strand breaks, resulting in lethal DNA damage (39). This effect potentially reduces the tissue radiosensitivity to oxygen tension (40–42). Under various particle beams, the OER for DSB induction and cell death was observed to decline with increased LET (40, 43–45). The Alpha–Howard–Flanders model and linear-quadratic (LQ) models were extended to fit the experimental data and take into account both oxygen level and particle LET (1, 43).

Our intention in this study is to abstract the concepts in oxygen depletion and vasculature re-oxygenation into a mathematical framework and seek a numerical proton FLASH radiobiological effect taking into account the dynamic oxygen level during delivery. The spatiotemporal oxygen level was the solution of a parabolic partial differential equation (PDE) that includes the radiation dose, delivery dose rate, metabolic depletion, and oxygen diffusion with vasculature oxygen supply boundary conditions. Adopting the conclusions from previous UHDR simulations that the tissue distance from the vessel (24, 34, 35) is vital for its initial and final stage of oxygen content, this study uses information of the local tissue vasculature spacing to confine the extent of diffusion distance. The local vasculature information can be related to tissue vasculature measurements and is different in various tissues. We proposed the 1D, 2D, and 3D solution space to the PDE, with confined boundary conditions from vessel oxygen supplies. As chaotic tumor vasculature development often results in lacunar features or blunt ends that affect the blood flow (46, 47), the affected oxygen supplies were built in as reduced oxygen supply boundary conditions (BCs). The simulation was performed in the stages prior to irradiation, dynamic radiation dose delivery, and after irradiation. For mice proton FLASH experiments, the experimental setup, including customized scatterers and collimation devices, were included in the Monte Carlo simulation to calculate the dose and the dose-weighted linear energy transfer LET_D_ (48). During each infinitesimal duration of the irradiation, the local oxygen level was obtained from the PDE solution; the impact to the delivered dose at the oxygen level was calculated from the proton hypoxic reduction factor (HRF). A cumulative normoxic-equivalent dose (CNED) was obtained for various irradiation situations. A FLASH effective-dose-modifying factor (FEDMF) derived as the ratio of CNEDs between CONV and UHDR deliveries was used to evaluate the FLASH effect. Once the characteristic vasculature structure of the irradiated tissue is known, the process can be adapted to correlate the dose delivery parameters to the outcomes of small animal UHDR biological experiments.

## Methods

### Monte Carlo dose and LET calculation

Monte Carlo simulation was used to obtain the 3D voxelized proton dose and LET distributions at the sites of the animal irradiation with a fast multi-core Monte Carlo package MCsquare (49, 50). This software simulates the dose and dose-weighted LET (LET_D_), which was experimentally validated (48, 51–53). The mouse CT DICOM images were modified to include the double-scattering animal proton FLASH platform (8). A 2-mm lead foil as the first scatterer, a lead ball bearing as the second scatterer, and an acrylic beam collimator with a brass collimation insert were built into the irradiated geometry. The mouse images or a water phantom was positioned downstream from these beam modifiers. The volume of the simulation is 100 × 15 × 15 cm^3^ re-sampled into 0.5 × 0.5 × 0.5 mm^3^ voxels. The proton pencil beam profile was experimentally characterized using a 2D transmission ion chamber IC-64 (Pyramid Technical Consultants, Boston, MA) with 1 mm resolution. A 2D Gaussian profile was used to fit the measured pencil beam profile (54) and input to the software as the beam source. The code ran on a Linux system equipped with dual 36-core CPUs and took ~6 h to finish a run with 10^9^ events. The 10^9^ particles used for this study is sufficient, as a robustness study of the MCSquare engine reportedly used 36 × 10^6^ particles to achieve a maximum statistical uncertainty of 1% (55). The simulated dose relative to the number of events was calibrated with an advanced Markus chamber. During the animal experiments, the dose rate increases linearly with cyclotron output current (8, 56), which was measured to be 213 ± 3 nA. A higher extracted current of 800 nA was recently reported on a hospital-based cyclotron (57). Our cyclotron has achieved similar extraction current previously (58) and was repeated recently, which produces 500 nA nozzle current. The time of the irradiation was recorded by an oscilloscope. The voxel-based dose, dose rate, and LET in mice were then obtained.

### Oxygen depletion and replenishment model

We used a mathematical model to simulate the dynamic oxygen level change in the tissue microenvironment, including the effects of irradiation, metabolism, and diffusion. The spatiotemporal oxygen distribution will be obtained as the numerical solution to a governing parabolic partial differential equation (PDE) factoring in the dose/dose rate and tissue types with characteristic vasculature structure. The radiation oxygen depletion rate was $\frac{\partial O}{\partial t}$ proposed to be proportional to the dose rates (24, 34, 35), where O is the oxygen concentration in the tissue. Here, we expressed the term as $\frac{\partial O}{\partial t}=-K_{1}\dot{D}\frac{O}{O+l}$ where $\dot{D}$ is the dose rate. The constant *K*_1_ represents the G-value of oxygen depletion. Radiolytic Monte Carlo simulation in deriving the oxygen G-value typically assumes abundant *in situ* oxygen supply (59, 60). However, at lower oxygen level, from the simulations by Lai et al. (61) and Boscolo et al. (62), the oxygen depletion rate reduces. The term $\frac{O}{O+l}$ reflects the reduced oxygen depletion rate at lower oxygen level with small *l* (0.29 μM from Lai et al.). Recently, measurements of proton radiation oxygen depletion in bovine serum albumin (BSA) solutions or Buffer 3G solution were made using phosphorescence quenching method (63) in addition to a similar measurement under electron beams (64). The G-value varies from 0.37 to 0.72 μM/Gy depending on the solution and dose rate. When considering the organic and labile ion processes, the radiation oxygen depletion rate is arguably much higher (22). In this study, we chose *K_1_ =* 0.5 μM/Gy.

The tissue vasculature provides essential oxygen for its biological functions. The oxygen diffuses transcapillary through the vasculature endothelial cells to tissues. We assume that within the time of interest in this model, the vasculature structure is stable with no radiation damage that alters its permeability or oxygen supply. Under normal physiological conditions, the oxygen level reduces as a function of radial distance from the wall (65, 66) governed by a PDE with metabolic and diffusion terms. The oxygen transport flux *via* tissue diffusion can be written as with diffusion constant *K*_3_ = 2×10^−5^ cm^2^/s (67). The tissue metabolic oxygen consumption (66) can be modeled by the Michaelis–Menten kinetics (68, 69) $\text{M}=-K_{2}\frac{O}{O+\lambda }$ . For high oxygen concentration, the oxygen is consumed by the tissue at an approximately constant rate *K*_2_. The rate of consumption rapidly decreases at lower hypoxic concentration (69, 70) regulated by the parameter *λ*. Combining the effects of radiation oxygen depletion, tissue oxygen diffusion, and tissue metabolic oxygen consumption, the oxygen concentration in tissue *O* is governed by a parabolic PDE:


(1) $$\frac{\partial O}{\partial t}=-K_{1}\dot{D}\frac{O}{O+l}-K_{2}\frac{O}{O+\lambda }+K_{3}\nabla^{2}O$$

In this study, we first simulated in 1D with dimension comparable to the vasculature spacing and $\nabla^{2}O=\frac{\partial^{2}}{\partial x^{2}}O(x,t)$ . The parameters of various dose/dose rates, oxygen distribution prior to irradiation, and oxygen boundary conditions were studied.

The parameters used for solving **Eq. (1)** are listed in **Table 1**. The initial condition of the oxygen concentration is the oxygen level at the start of the irradiation and is regarded as dynamically balanced between oxygen metabolism and diffusion. The 1D simulation space is bounded by two blood vessels at the ends. The blood vessels provide constant oxygen supply with Dirichlet boundary condition (BC) as, *O* ( $\vec{x},t$ )|_∂Ω_ = *O_v_* where Ω is the simulation space. The tumor structure is heterogeneous with lacunar features or blunt ends where highly hypoxic regions exist from low blood oxygen supply of these vessels (47). Healthier vasculature provides a larger blood oxygen supply. In this study, we simulated BC from 5.4 to 50.4 μM.

**Table 1:** Parameters used in solving PDE **Eq. (**1**)**.

	Parameter	Values
Radiation oxygen depletion rate	*K_1_, l*	0.5 μM/Gy, 0.29 μM
Oxygen metabolism	*K_2_, λ*	18.9 μM/s and 3.15 μM
Tissue oxygen diffusion	*K_3_*	2×10^−5^ cm^2^/s
Oxygen boundary condition	*O_v_*	2.4–50.4 μM
Inter-vessel distance	*Ω*	25–1,000 μm
Dose rate	$\dot{D}\left(\vec{x},t\right)$	CONV 0.5 Gy/s, UHDR 125 and 285 Gy/s
Dose	*D*	2, 8, 15 Gy

The simulation space of the PDE was chosen as equivalent to the vasculature spacing. Scanning electron microscope stereo-imaging (SEM) and micro-computed tomography (micro-CT) had been used to characterize the vasculature structures and revealed that the vasculature of certain normal and tumor tissues have inter-vessel distances within the range of 25–800 μm (Konerding et al., 1999, Folarin et al., 2010). The skin vasculature could have larger vessel spacing (71). We chose a representative selection of the inter-vessel spacing at 25–1,000 μm to solve the PDE in **Eq (1)**. Therefore, the simulation space Ω is confined between two vessels with defined spacing.

The dose rate $\dot{D}$ was applied as a step function in time.


$$\dot{D}=\{\begin{array}{cc} 0 & t<0\\ \dot{D}\left(\vec{x},t\right) & 0<t<T\\ 0 & \;t>T \end{array}.$$

Here *T* is the irradiation duration. The total delivered dose is $D\left(\vec{x}\right)=\int_{0}^{T}\dot{D}\left(\vec{x},t\right)dt$ . In this study, the dose rate was assumed independent of the spatial variable $\vec{x}$ and irradiation time *t*, $\dot{D}={\dot{D}}_{0}$ . In typical patient treatment plan, dose is calculated at CT voxel level with the dimension of 1–3 mm. Typical vasculature spacing is smaller than voxel dimension. Therefore, within the vasculature spacing, we assume a constant dose rate. Before irradiation t<0, the PDE was solved until a dynamic equilibrium state between metabolism and oxygen diffusion was achieved; that is, the change in oxygen level at each time step was less than 10^–4^ *O*. The oxygen distribution *O* ( $\vec{x},0$ ) was used to solve for the irradiation time period 0 < *t* < *T* and then oxygen replenishment at *t* > *T* following the dose rate step function. Each spatial–temporal solution at the stages prior to radiation, during radiation, or post-radiation was obtained with 1,500 time steps. Then, 3,000 time steps for each stage for a selected subset of initial and BCs were used to rerun the simulation and verify the stability of the solutions.

**Figure 1:**
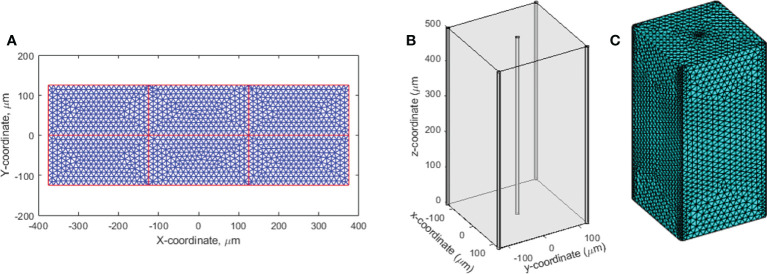
2D and 3D geometries for oxygen distribution solution to Eq (1). **(A)** The 2D region to simulate the dynamic oxygen level changes under various dose rates. The vessels across the region are shown in red color. The triangular mesh is illustrated and shown in 10 times of its actual size. **(B)** The 3D cuboid geometry. Five vessels across the region at the center and four vertical edges concurrently provide oxygen supplies. **(C)** The 3D mesh.

The oxygen depletion and replenishment model can be extended to 2D and 3D solution space using finite element method to solve **Eq (1)**. We demonstrated the 2D solution to a region where multiple vessels cross and concurrently provide oxygen supply. As shown in **Figure 1A**, the 2D rectangular region was surrounded with four vessels. Within the region, seven other vessels represented the capillary network crossing the region. Each of the subdomains in the 2D region was meshed with triangular elements. The Dirichlet boundary condition was applied to the surrounding and capillary network vessels as oxygen supply sources to the region. The time-dependent nodal solution to **Eq. (1)** in the region was solved under CONV and UHDR dose deliveries.

The finite element method was used to solve **Eq. (1)** in the 3D solution space described in **Figure 1B**. The cuboid has five vessels passing through the region. One is at the center and the other four at the four vertical edges. Tetrahedral mesh elements were applied (**Figure 1C**). Finer mesh was generated at the interface to the vessels. At the vessel interfaces, Dirichlet BCs were set as *O*( $\vec{x},t$ )|_∂Ω'_ = *O_v_*, where ∂Ω' represents the interfaces to the vessels. Newmann BCs ∇*O*( $\vec{x},t$ )^•^ $\vec{n}$ |_∂Ω"_ = 0 were set to other surfaces ∂Ω" as the cuboid is assumed to repeat itself in space, and the solution is therefore symmetric at the surfaces.

### Proton FLASH effective-dose-modifying factor

The oxygen enhancement ratio (OER) was used to indicate the effective dosimetric response or tissue biological response under different tissue oxygen concentrations. The tissue radiosensitivity is a function of tissue oxygen concentration and particle LET, and its value declines with increasing LET (39, 44). The particle oxygen radiosensitivity was found to have small dependence on cell type and the delivered dose; when using LET_D_, the oxygen radiosensitivity is independent of the ion type (1, 72). Scifoni et al. (1) studied the dependence of the survival level as a bidimensional parameterization of LET_D_ and oxygen. In this paper, we used the same parameterization formula and the term hypoxia reduction factor (HRF) (65) as the ratio of the dose at given oxygen level and LET to the dose at normoxic condition that produces the same survival level.


(2) $$HRF\left(LET,O\right)=\frac{D\left(LET,O\right)}{D_{norm}}{|}_{same\;survival}=\frac{b\left(aM+LET^{\gamma }\right)/\left(a+LET^{\gamma }\right)+O}{b+O}$$

where M is the anoxic condition of maximum effect (~3); *γ* =3; and *a*=8.27×10^5^ (keV/μm)*^γ^* and *b*=2.4 μM. In this equation, the LET_D_ distribution of the proton was input from the small animal Monte Carlo simulation, which is time independent for the scattered-field dose delivery. The oxygen distribution is the numeric solution from Eq. (1).

The derived *HRF*( $\vec{x},t$ ) from Eq. (2) is spatiotemporally distributed within the vasculature intraspacing. At the end of the irradiation, we define the cumulative normoxic-equivalent dose (CNED) as the cumulative dose equivalent to achieve the same biological effect under normoxic condition. Using the definition of HRF, CNED can be expressed as

$CNED\left(\vec{x}\right)=\int_{0}^{T}\frac{\dot{D}\left(\vec{x},t\right)}{HRF\left(\vec{x},t\right)}dt$


for constant dose rate (3) $=\dot{D}\int_{O}^{T}\frac{1}{HRF\left(\vec{x},t\right)}dt$

The *CNED* is the output of this multi-stage model framework, which includes the effects of ion beam characteristics and dynamic oxygen effect. As the tissue HRF≥1, CNED is smaller than the delivered dose *D*. The dynamic oxygen effect from the irradiation can be expressed by the ratio of the two *CNED*( $\vec{x}$ )/*D*. To characterize the FLASH sparing effects, Bourhis et al. proposed using the FLASH dose-modifying factor to indicate the ratio of the dose delivered in CONV and UHDR dose rates to achieve the same biological effect (11). They summarized that >1.3 dose-modifying factor were observed in various *in vivo* experiments. Using a similar concept, we defined the proton FEDMF in this study as the ratio of *CNED*( $\vec{x}$ ) the under CONV and UHDR dose rates and considered the FLASH effect with FEDMF*≥*1.20,


(4) $$FEDMF\left(\vec{x}\right)=\frac{CNED{\left(\vec{x}\right)}_{CONV}}{CNED{\left(\vec{x}\right)}_{UHDR}}$$

## Results

The Monte Carlo simulation software MCSquare was used for mice irradiation dose and LET_D_ calculation. From the beam profile, a 20%–80% penumbra of 7.3 mm was obtained in mice, which is consistent with the film profile measurement. The irradiation ultra-high dose rate was calculated as 126.5 Gy/s and 285 Gy/s at 222 and 500 nA nozzle current. The LET_D_ at the mice was derived as 1.2 keV/μm.

**Figure 2:**
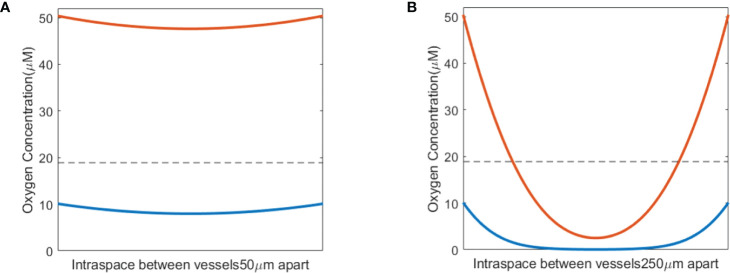
Pre-irradiation oxygen levels taking into account oxygen metabolism and diffusion. **(A)** Small vasculature spacing results in oxygen levels similar to vasculature oxygen BCs—normal BC of 50.4 μM (orange) or poor BC of 10.1 μM (blue). **(B)** Hypoxic regions develop between blood vessels in conjunction with large vasculature spacing, shown for both normal (50.4 μM; orange) and poor (10.1 μM; blue) oxygen BCs. The dashed line in panels **(A, B)** indicates the hypoxic threshold of 18.9 μM.

The oxygen level before irradiation (*t<*0) was examined first at various inter-vessel spacing. The inter-vessel oxygen spatial solution shows that at fixed vascular oxygen supply BC, a dynamic equilibrium oxygen status is maintained between the metabolic consumption and diffusion. Poor BC in the tumor environment results in a hypoxic region (using hypoxic threshold 18.9 μM in this study) throughout the vessel spacing (blue curves in **Figures 2A, B**). In a healthy tissue with normal vasculature BC, a larger vessel spacing causes a hypoxic central region at a distance from the vessels (orange curve in **Figure 2B**). Comparing the two curves in **Figure 2B**, poor BC and large vasculature spacing create a severe hypoxic region, while a healthy BC and large vessel spacing result in a more moderate hypoxic region between the vessels. Both low BC or large vessel spacing can result in a hypoxic region. For example, a hypoxic region starts to appear at 175 μm spacing and 50.4 μM BC, and at 125 μm spacing and 35 μM BC. At 250 μm spacing and 50.4 μM BC, 45% of the space between vessels is hypoxic with a mean oxygen level of 24.1 μM.

**Figure 3:**
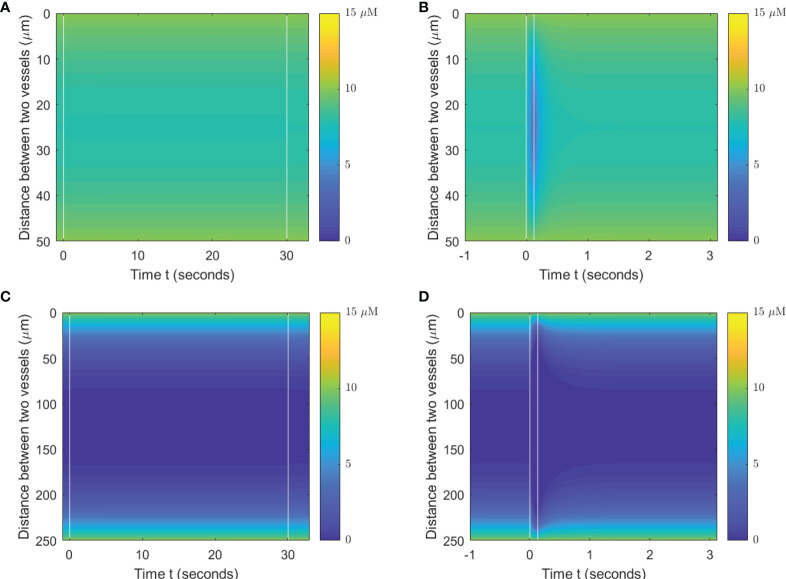
Spatiotemporal changes in oxygen levels for different vasculature spacings and dose rates. Oxygen levels (μM) under 15 Gy irradiation with spacings of 50 μm **(A, B)** and 250 μm **(C, D)** under BC=10.1 μM. The time between two vertical white lines indicates the duration of the irradiation. **(A, C)** Irradiated with 0.5 Gy/s; **(B, D)** irradiated with 125 Gy/s.

**Figure 4:**
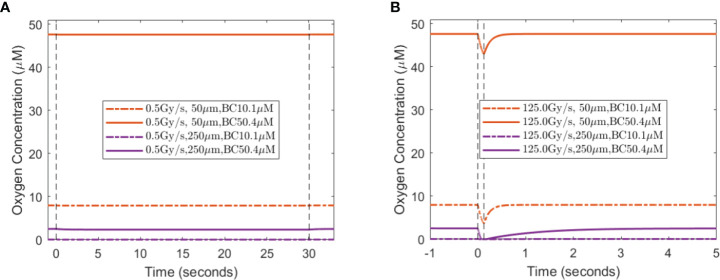
Midpoint oxygen levels for different oxygen supply BCs, vasculature spacings, and dose rates. The change in oxygen concentration from 15 Gy irradiation under the dose rates of **(A)** 0.5 Gy/s and **(B)** 125 Gy/s under 10.1 and 50.4 μM for 50 and 250 μm spacings. The dashed vertical lines indicate the duration of the irradiation.

When a single-field dose is delivered at CONV and UHDR dose rates, 0.5 and 125 Gy/s, it will result in different spatiotemporal oxygen depletions, the extent of which depends on the vasculature spacing and BC. **Figure 3** shows the spatiotemporal oxygen levels as the solution to **Eq. (1)** pre-, during, and post-irradiation of 15 Gy under CONV and UHDR deliveries with two vasculature intraspacings and 10.1 μM BC. The changes in oxygen levels at the mid-point between the vessels are shown in **Figure 4**. **Figures 3A–C**, **4A** show that regardless of the vasculature spacing and BCs, CONV dose rate creates minimal changes in the oxygen concentration. This result infers that for all tissue types under CONV dose rate, the effect from radiation-induced oxygen depletion is negligible. UHDRs can generate reduced oxygen level before oxygen replenishment from nearby vessels occurs, as shown in **Figures 3B–D**, **4B**, which can be responsible for the radiation-induced FLASH radioprotection effect. At lower BC *O_v_* =10.1 μM, the oxygen depletion by fast dose rate is apparent for small vasculature intraspacing of 50 μm, especially in the central region of the vasculature spacing (**Figure 3B**). **Figure 4B** (dashed orange line) shows that the oxygen level at the mid-point drops from 8.0 to 3.7 μM after the irradiation. At larger vasculature spacing of 250 μm, the originally severe hypoxic central region does not experience much further oxygen depletion, regardless of the dose rates (**Figures 3C, D**). In this case, the mid-point oxygen level remains<0.1 μM (dashed purple lines in **Figures 4A, B**). Under ultra-high dose rates, a healthy vasculature (BC=50.4 μM) experiences a reduction in oxygen concentration with radiation dose in both large and small vasculature inter-vessel space. At the midpoint, the oxygen level is reduced from 47.6 to 43.0 μM for 50 μm spacing and 2.5 to 0.0 μM for 250 μM spacing.

A lesser dose causes smaller changes in oxygen levels under the same high dose rate. At the midpoint of 50 μm spacing, 2, 8, and 15 Gy reduce the oxygen level by 0.8, 2.8, and 4.3 μM, respectively, with 10.1 μM BC, and 0.8, 2.9, and 4.7 μM, respectively, with 50.4 μM BC. The amount of oxygen level reduction is smaller at the midpoint of 250 μM spacing. For 2, 8, and 15 Gy irradiation, the changes in oxygen concentration are 0.0, 0.1, and 0.1 μM, respectively, with 10.1 μM BC, and 0.7, 2.3, and 2.5 μM, respectively, with 50.4 μM BC. After irradiation, the oxygen level recovers from radiation-introduced depletion within a few seconds.

**Figure 5:**
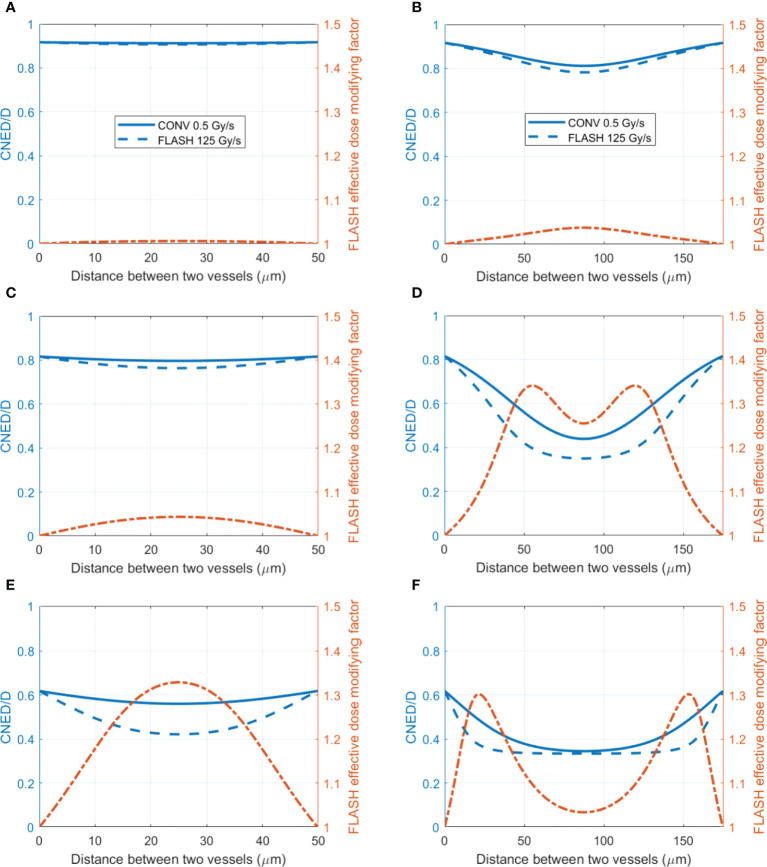
The ratio of CNED to delivered dose D and FLASH effective-dose-modifying factor for different vasculature intraspacings and oxygen BCs. (Left axis) The ratio of the CNED to the delivered 15 Gy dose under conventional 0.5 Gy/s (solid blue) and UHDR 125 Gy/s (dashed blue) dose rates; (right axis) the FLASH effective-dose-modifying factor derived from the CNEDs under the two dose rates. Two different vasculature spacings [**(A, C, E)** 50 μm; **(B, D, F)** 175 μm] with high 50.4 **(A, B)**, medium 18.9 **(C, D)**, and low 5.4 μM **(E, F)** BCs are shown.

**Figure 6:**
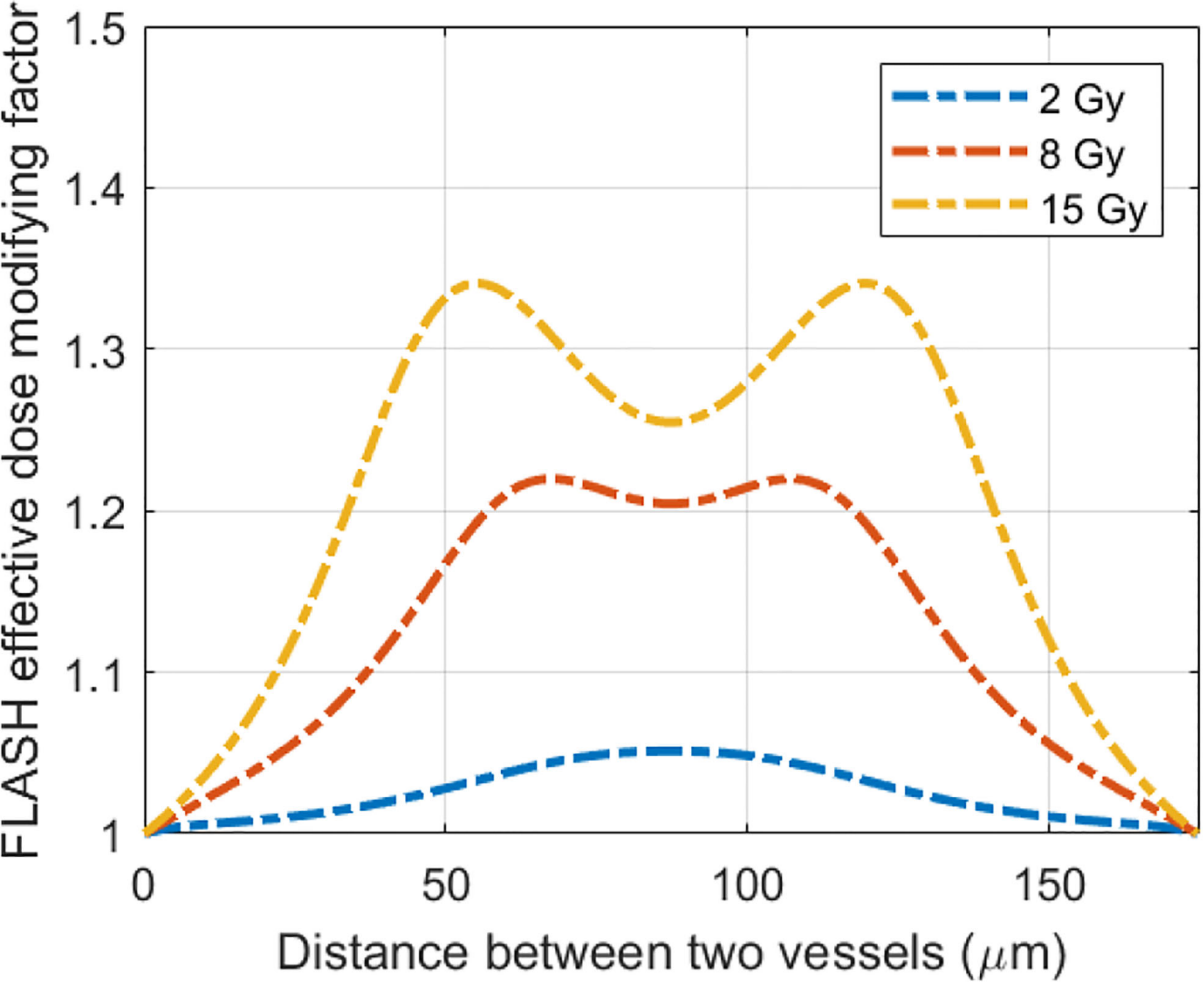
The delivered dose makes a difference in the FLASH effective-dose-modifying factor. Plotted are the FLASH effective-dose-modifying factors from 15 Gy delivered at 0.5 and 125 Gy/s dose rates for 175 μm vasculature spacing and 18.9 μM BC.

Based on the amount of oxygen depletion by CONV and UHDR dose rate, the dynamic HRF and CNED can be derived using **Eqs. (2)** and **(3)** **Figure 5** shows cases of the radiation oxygen depletion effect using the dose ratio *CNED*( $\vec{x}$ )/*D* (blue curves) under 0.5 and 125 Gy/s with 15 Gy against the left y-axis. The FEDMFs as the ratio of the CNEDs under CONV and UHDR dose rates are plotted in orange against the right y-axis. As shown previously, FLASH dose delivery further depletes the oxygen compared with the CONV delivery. The $CNED{\left(\vec{x}\right)}_{UHDR}$ everywhere in the space is smaller or equal to the $CNED{\left(\vec{x}\right)}_{CONV}$ , which results in FEDMF≥1. The differential FLASH effect between CONV and UHDR deliveries can be inferred from the FEDMF. From **Figure 5**, the impact to the FLASH effect from the vasculature spacing and BC is apparent. At well-oxygenated 50.4 μM BC, radiation within both 50 and 175 μm vasculature spacings result in very small oxygen effects regardless of the dose rates (**Figures 5A, B**). Hence, the FEDMF is close to unity, indicating no apparent FLASH effect. At an intermediate BC of 18.9 μM, small vasculature spacing does not show the FLASH effect where the FEDMF is close to 1, due to the short distance from the nearby vessels and quick replenishment of depleted oxygen. However, within the pre-existing hypoxic region prior to irradiation at 175 μm vasculature spacing, FLASH-introduced radio-protection effect appears with apparent differential CNEDs between UHDR and CONV dose deliveries. It results in 61.5% of the inter-vessel space with larger than 1.20 (or 20%) FEDMF (**Figure 5D**). Under poorer oxygen BC of 5.4 μM, differential FLASH effects show up in both 50 and 175 μm, but to a much less extent compared with 18.9 μM BC, which has 55.0% and 31.0% space with FEDMF above 1.20. If the intermediate BC can represent the situation in normal tissues, the FLASH effects are more prominent than the poor BC cases, as in the tumor environment. When considering different dose deliveries, the amount of region with FEDMF≥1.20 reduces with delivered dose (**Figure 6**). The percentage of the space with FEDMF≥1.20 reduces from 61.5% for 15 Gy to 34.5% and 0% for 8 and 2 Gy deliveries in the 175 μm vasculature spacing.

**Figure 7:**
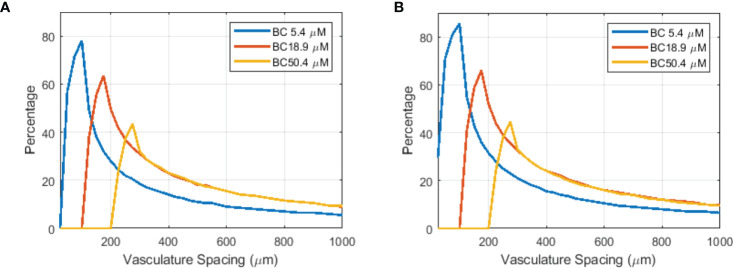
Flash effective-dose-modifying factor relates to vasculature spacing, oxygen BCs, and FLASH dose rates. Percentage of the region with FEDMF≥ 1.20 is plotted against the vasculature spacing for different BCs (5.4, 18.9, and 50.4 μM) for two different proton FLASH dose rates: **(A)** 125 Gy/s and **(B)** 285 Gy/s.

Although limited blood oxygen supply is typical for tumor vasculature (73), vasculature spacing is not well characterized for various normal and tumor tissues. In one study by Folarin et al. (74), the most probable vasculature spacing appears to be ~100–200 μm in both colorectal normal mucosa and tumor tissues. Our simulation shows that at 175 μm, better oxygenated normal tissue shows a larger region (61.5% for 18.9 μM BC) with FEDMF≥1.20 than poorer oxygenated tumor tissue (31.0% for 5.4 μM BC). For other vasculature spacings, **Figure 7A** plots the percentage of the region with FEDMF≥1.20 under 5.4, 18.9, and 50.4 μM BCs. At smaller than 100 μm vasculature spacing, the percentage of the region with FEDMF≥1.20 can be larger than 50% in poor BC of 5.4 μM. This percentage quickly reduces at larger vasculature spacing. The FLASH effect does not show up for 18.9 μM BC unless with larger than 100 μm vasculature spacing and peaks at 175 μm. At larger than 125 μm inter-vessel spacing, 18.9 μM BC has a larger region with FEDMF≥1.20 than the smaller 5.4 μM BC. It could indicate that the FLASH normal tissue sparing should be more readily observable in tissues with larger vasculature spacing.

Higher dose rate increases the amount of the region with FLASH effects. At an increased dose rate of 285 Gy/s with ~800 nA cyclotron output current, a larger percentage and wider range of the vasculature spacing will show the FLASH effect (**Figure 7B**). This could potentially indicate that the FLASH effect would be more readily observed in tissues with wider range of vasculature spacing when the UHDR dose rate increases.

We also examined the FLASH effects under irradiation with higher LET_D_ near the Bragg peak (BP). At LET_D_ of 10 and 20 keV/μm of 15 Gy to 175 mm vessel spacing, 61.5 and 61.0% of the region shows FLASH effect for BC of 18.9 μM, and 31.0% and 31.0% for BC of 5.4 μM. It is a minimal change from the low LET deliveries. FLASH effect from other vessel spacing and BCs showed the same independence of the proton LET. This result is reasonable, as the summarized experimental data (1, 43) demonstrated that the difference in cell survival introduced by LET only occurs at much higher LET, for example, in carbon ion beams. This result is validated by a recent FLASH study on mice (75), which showed that dose from entrance and SOBP under ultra-high dose rates both preserved a significantly higher number of crypt cells and demonstrated similar tumor growth compared to the deliveries under conventional dose rate.

**Figure 8:**
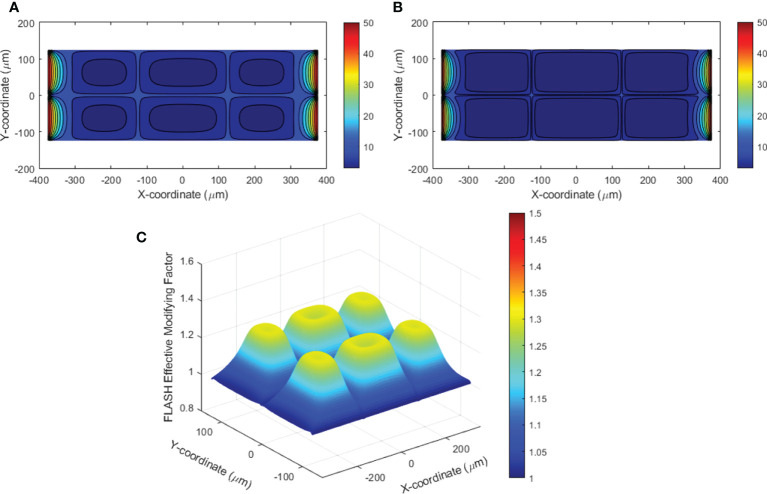
**(A)** Contour plot of the oxygen distribution in the simulated 2D region prior to irradiation; **(B)** contour plot of the oxygen distribution after UHDR irradiation of 10 Gy at 285 Gy/s; **(C)** FLASH effective-dose-modifying factor (FEDMF) comparing the CONV and FLASH effective dose using the hypoxic reduction factor model.

An example of the dynamic oxygen simulation in 2D space was performed in a 750×250 μm^2^ rectangular region as shown in **Figure 1**. In this finite element model, 227,104 elements and 557,832 nodes were generated. Well-oxygenated supply of 50.1 μM was provided at the left and right boundaries. The other vessels provided 10.1 μM BCs. A dose of 10 Gy at 0.5 and 285 Gy/s dose rates were simulated. **Figure 8A** demonstrates the oxygen distribution prior to irradiation from the balance of vessel oxygen supplies and metabolic consumption. The dose delivered at CONV dose rate minimally altered the oxygen distribution in the region. However, the dose delivered at FLASH dose rate further reduced the regional oxygen level (**Figure 8B**). The time-varying oxygen nodal solution was used to calculate the FLASH effective-dose-modifying factor FEDMF using **Eqs (2)–**(4). **Figure 8C** shows the FEDMF distribution in the region. It is observed that substantial portion 58.5% of the region demonstrates FEDMF*≥*1.20.

**Figure 9:**
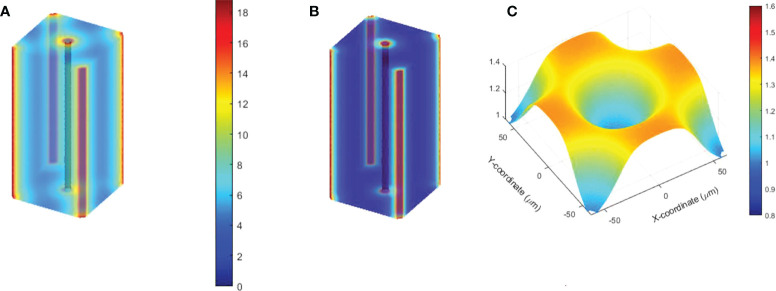
Semi-transparent 3D plots of the oxygen distribution under 15 Gy at **(A)** 0.5 Gy/s and **(B)** 125 Gy/s. The distance from the center vessel to the edge vessels is 88.4 μm. **(C)** The resulting FEDMF in the cross-section plane at half of the cuboid height.

The oxygen distribution solutions in 3D space were obtained under various vessel oxygen supply and dose delivery conditions. **Figures 9A, B** show the oxygen distributions after 15 Gy at 0.5 and 125 Gy/s irradiations, where the vessels supply 18.9 μM BCs. The region away from vessels shows differential oxygen level under CONV than under UHDR irradiations, although both are hypoxic. According to **Eqs (2)–**(4), the voxel CNEDs are different under different dose rates and results in 73.6% of the 3D volume showing FEDMF≥1.20. **Figure 9C** shows the FEDMF in the cross-section plane at half of the cuboid height. In the region where CONV and UHDR hypoxic levels are different, high FEDMF values are shown. Higher dose rate will increase the region of high FEDMF. At 285 Gy/s, 78.4% of the 3D volume shows FEDMF≥1.20. Under 2, 8, and 10 Gy irradiations of the same geometry and BCs, the percentage volume showing FEDMF≥1.20 reduces to 0%, 0%, and 16.6% compared with 0.5 and 125 Gy/s. When the vessels are further apart, the percentage volume showing FEDMF≥1.20 also reduces. For 15 Gy, it reduces to 72.1% and 23.5% when the center vessel is 123.7 and 176.8 μm, respectively, from the edge vessels.

## Discussion

In this study, we modeled the radiation oxygen depletion process with the proton FLASH dose delivery parameters in 1D, 2D, and 3D space. The tissue vasculature spacing parameter, which can be quantified for normal and tumor tissues (74, 76), was introduced as the PDE solution space. We demonstrated that prior to irradiation, larger vasculature spacing causes more hypoxic regions in tissues even under healthy vasculature oxygen supply BCs. This is consistent with the experiments summarized by Hendry (25) that demonstrated the possible existence of hypoxic normal tissues in the skin, lung, and gut. If the normal tissue is considered in a uniform physioxia stage, as Pratx and Kapp (24) pointed out, the <1 μM/Gy radiation oxygen depletion rate will not demonstrate FLASH sparing effects for >50.4 μM oxygen concentration. They pointed out that hypoxic stem cell niche exists in normal tissues, which experience the FLASH radioprotection effect (77). We also showed that the pre-existing hypoxic region is responsible for a smaller CNED under UHDR comparing with that under CONV dose rate. When the initial hypoxic level is at the steep gradient region of the HFR curve, under high dose rate irradiation, the radiolysis and ROS reactions cause the oxygen level to quickly go down further before the replenishment from nearby blood vessels can occur. The infinitesimal accumulation of the HFR-weighted dose shows a reduced dosimetric effect. This effect does not occur when the pre-existing hypoxic condition is severe, for example, under larger vasculature spacing and poor BCs. Our simulation results confirmed the previous studies with single-vessel as the oxygen source (24, 35) that the distance from the vessels is essential in providing FLASH sparing effects. We further demonstrated both in 1D and 3D solutions that the oxygen supply BCs are crucial to the FLASH effects. As shown in **Figure 6**, at large vasculature spacing, healthier oxygen supply BC will show up more FLASH effects compared with poorer oxygen BC. This result supports the observation in skin FLASH experiments in animals and patients (6, 14) where skin has typically larger vasculature spacing between two plexuses (71).

The vasculature information is not readily available for all the normal and tumor tissues interested to the clinic or used in *in vivo* UHDR experiments. Literature survey shows few tissues were characterized for their vasculature structures (71, 74, 76, 78, 79). In addition, the blood oxygen supply is dependent on the vessel endothelial structure, diameter, and distance from the major vessel where the measurements and dynamic simulations were performed in few selections of tissues (68–70, 80). It involves the disassociation of the oxygen from the blood cells and perfuses through the permeable vessel walls of epithelial cells. The vessel oxygen supply also varies due to the autoregulation of the blood flow caused by activities (81) and could react to the reduction by the radiation. Typical stress-induced functional recruitment (82) can elevate the blood oxygen content by 6%–20% with a time constant about 10 s (83). In this model, it is possible to apply a transient vessel oxygen BCs to simulate the functional recruitment, *0*( $\vec{x},t$ )|_∂Ω_ = *0_v_* (1 + αerf(2*t*/*T*)) where α is the vessel oxygen elevation level and T is the time constant. Using α=20% and T=10 s, the oxygen level under UHDR irradiation does not change much compared with the results using constant BC, due the ultra-fast delivery. The oxygen level under CONV irradiation appears slightly higher than the solution with constant BC, which results in larger differential effective dose between the UHDR and CONV deliveries, therefore increased FEDMF. In an example of 175 μm inter-vessel space and 18.9 μM BC, transient BC results in 68.5% of inter-vessel space with FEDMF ≥ 1.20, 7% more than the situation when constant BC is considered. Future experiments on the vessel blood flow and transient oxygen supply under irradiation condition would be of great interest to understand the tissue oxygen levels. Furthermore, we realized that the local vasculature of tumor and normal tissues is a complex 3D structure. The 1D solution space is an oversimplified model that has unrealistic assumptions on the geometry. It must use the dose delivery parameters and oxygen depletion rate from 3D simulations or measurements to have meaningful results by using a space of same dimension. We have demonstrated that with finite element method, the solution space can be extended to 2D and 3D regions with multiple vessels across the region and provide oxygen concurrently. It is possible to obtain the oxygen depletion solution within a realistic tissue/vasculature 3D space with finite element method. Cui and Pratx (84) provided an example of mesh in 3D vasculature space in a mouse brain and worked on oxygen level recovery after the irradiation. To extend our current work into 3D vasculature space, it is imperative to perform the simulation on a representative collection of the animal tumor and normal tissue vasculature models to derive statistically meaningful results for FLASH sparing effects.

In this paper, we have used constant *K_1_* for both conventional and FLASH oxygen depletion rates. As several recent publications (33, 85, 86) pointed out, at very high track density per unit time under ultra-high dose rate, radical chain reactions occur inter-track, which can potentially reduce the oxygen consumption. *In vitro* measurements of oxygen depletion using phosphorescence quenching method (64, 87) supports reduced oxygen depletion rate under ultra-high dose rate compared to that under the conventional dose rate. Further study is needed to understand the *in vivo* oxygen depletion rate under different modalities in both normal and tumor tissues and make necessary adjustment to this model.

In this study, we showed that the local FEDMF varies throughout the inter-vessel spacing. The percentage of the inter-vessel spacing with calculated FEDMF≥1.20 was used to quantify the FLASH effect. As shown in **Figure 4**, these regions are at a distance away from the vessels where the oxygen replenishment by diffusion from vessel is not instantaneous, so that FLASH oxygen depletion causes a local oxygen level different from that in the CONV delivery. Currently, it is not clear how much the percentage has to be in order for the FLASH sparing radiobiological outcome to be observed in different tissues. To establish the relationship, the animal tissue vasculature characteristics in terms of vessel spacing and vessel oxygen supplies should be experimentally characterized. Then, a series of UHDR and CONV studies with various levels of delivery parameters and defined biological endpoints should be carried out to correlate with the simulation results. Nonetheless, we have shown that under current clinical standard fractionation of 2 Gy/fraction delivery, no region shows FEDMF≥1.20. Therefore, it is very likely that 2 Gy FLASH deliveries will not exhibit any FLASH sparing effect. Even at 8 Gy delivery, it is questionable that the FLASH sparing effect can be observed experimentally. In our simulation, higher dose rates and delivered dose increase the percentage of the region showing significant FEDMF, which could indicate that the FLASH radiobiological effect is more readily observable under higher dose rates.

In this paper, we simulated oxygen depletion as a generalized concept beyond the individual cell level. The reduced radiosensitivity under hypoxia used in AHF or particle HRF models were adopted from experimental *in vitro* cell experimental data. One caveat in using the particle HRF model is that these data did not include the dose rate information, which should be considered in the future for particle FLASH HRF model. In addition, the oxygen level was generally measured extracellularly. On the other hand, microenvironment oxygen depletion simulations have been performed using the mechanism of oxygen scavenging electrons and hydrogen atoms in pure water as the model for ROD (61, 88, 89). It is worth pointing out that other oxygen scavengers in tissues such as peroxyl radicals and the reaction chain can, in turn, re-supply the oxygen (33, 86, 90). As the oxygen level is different at extracellular and at nucleus level, establishing the relationship of cell level oxygen solubility and the magnitude of radiation oxygen depletion under different dose rates can help with the understanding of the radiation oxygen-depletion mechanism.

For higher LET beams, FLASH effect due to oxygen depletion mechanism will decrease. This is due to more direct damage to DNA than indirect damage involving free radicals. For carbon ion beam of 60 MeV/u from plateau to Bragg peak region, the LET values vary from 50 to >400 keV/μm (91). Using our 1D model, at the plateau region, 15 Gy with LET as high as 50 keV/μm changes the region showing FEDMF≥1.20 to 59.5% (with BC of 18.9 μM, 175 μm vessel spacing), a 2% reduction from the proton irradiation. This value drops drastically to 22% at LET of 100 keV/μm, to 0% at LET of 200 and 400 keV/μm towards the Bragg peak. Therefore, minimal FLASH sparing effect is expected at the carbon ion Bragg peak region. In addition, it is considered that an oxygenated microenvironment exists from multiple ionization of water for high LET particles (41), which can reduce the local effective oxygen depletion rate and achieve more efficient cell killing. The simulation by Zakaria et al. (92) pointed out that UHDR carbon ion beams can realize the FLASH sparing of normal tissues at the plateau region and efficient tumor control at the Bragg peak region.

Although the oxygen depletion under high dose-rate radiation is one of the hypotheses for the FLASH sparing effect, other mechanisms were proposed. Indeed, when a certain region of the tumor has optimal vasculature spacing and oxygen supply, it can potentially receive the FLASH sparing effect, based on the oxygen depletion model in this work. However, anti-tumor efficacy has been observed to be the same regardless of the dose rate. As many pointed out, FLASH oxygen depletion mechanism can only be one of the FLASH mechanisms that synergistically realize the FLASH sparing effect in normal tissue and equipotent control of tumor growth. Immune response activation can also be responsible for the FLASH effects (13, 93). The modified response from UHDR beams is evidenced by the amount of the induction of the transforming growth factor beta (TGFβ) signaling cascade (3, 18, 19). Differential vessel response between UHDR and CONV deliveries also occurs (19, 94). The investigation of the FLASH radio-biological mechanism(s) is still an ongoing task for the radio-biology community.

## Conclusion

We demonstrated in this study an integrated simulation approach of the UHDR oxygen depletion effect for small animal proton irradiation experiments. The local tissue vasculature spacing and oxygen supply are important in setting different tissue oxygen levels prior to irradiation. With the local vasculature information, we investigated the FEDMF as an indicator of the FLASH effect under various proton dose and dose rate levels. The amount of the inter-vessel space showing significant ≥20% FEDMF was derived. Larger dose and higher dose rate can result in a more pronounced FLASH effect. The FLASH effect was not found to be sensitive to the proton LET_D_ in the context of this modeling framework. The vasculature spacing and blood oxygen supply are quantifiable and have been performed in a few types of tissues. With more tissue-specific vasculature information, future work will include the correlation of the simulation results with FLASH biological experimental results, which will eventually help the translation of the UHDR delivery technique to the clinic.

## Data availability statement

The original contributions presented in the study are included in the article/supplementary material. Further inquiries can be directed to the corresponding author.

## Author contributions

WZ, ED, LD initiated the concepts for simulation. KH, WZ conducted the body of the work. RW, TB, MK performed the experiments. DC, YF, CaK, CoK, YX, KT, TB, KC, JM, AM contributed the content in the simulation. All authors contributed to the article and approved the submitted version.

## Funding

This work is supported by NIH 1P01CA257904-01A1.

## Acknowledgments

The authors would like to thank Dr. Sumin Zhou for going over the manuscript and providing valuable suggestions, and Dr. Kevin Souris and Dr. Sheng Huang for their help setting up MCSquare.

## Conflict of interest

The authors declare that the research was conducted in the absence of any commercial or financial relationships that could be construed as a potential conflict of interest.

## Publisher’s note

All claims expressed in this article are solely those of the authors and do not necessarily represent those of their affiliated organizations, or those of the publisher, the editors and the reviewers. Any product that may be evaluated in this article, or claim that may be made by its manufacturer, is not guaranteed or endorsed by the publisher.
